# A novel compound heterozygous variant of *ECEL1* induced joint dysfunction and cartilage degradation: a case report and literature review

**DOI:** 10.3389/fneur.2024.1343025

**Published:** 2024-01-24

**Authors:** Siyuan Jing, Mou Peng, Yuping He, Yimin Hua, Jinrong Li, Yifei Li

**Affiliations:** ^1^Key Laboratory of Birth Defects and Related Diseases of Women and Children of MOE, Department of Pediatrics, West China Second University Hospital, Sichuan University, Chengdu, China; ^2^Department of Nursing, West China Second University Hospital, Sichuan University, Chengdu, China

**Keywords:** *ECEL1*, joint disorder, WES, case report, literature review

## Abstract

**Background:**

Distal arthrogryposis type 5D (DA5D) represents a subtype of distal arthrogryposis (DA) characterized by congenital joint contractures in the distal extremities. DA5D is inherited in a rare autosomal recessive manner and is associated with the *ECEL1* gene. In this report, we describe a case of an infant with bilateral knee contractures and ptosis, caused by a novel compound heterozygous mutation of *ECEL1*.

**Case presentation:**

We conducted DNA extraction, whole-exome sequencing analysis, and mutation analysis of *ECEL1* to obtain genetic data on the patient. We subsequently analyzed the patient’s clinical and genetic data. The proband was a 6 months-old male infant who presented with significant bilateral knee contracture disorders and bilateral ptosis. MRI demonstrated cartilage degradation in knee joint. Whole-exome sequencing of the patient’s DNA revealed a compound heterozygous mutation of c.2152-15C>A and c.110_155del in *ECEL1*. Analysis with the MutationTaster application indicated that c.110_155del was pathogenic (probability = 1), causing frameshift mutations affecting 151 amino acids (p.F37Cfs*151). The truncated protein lost the substructure of a transmembranous site based on the predicted protein crystal structure AF-O95672-F1. The variant of c.2152-15C>A of *ECEL1* was also predicted to be disease-causing (probability = 0.98) as it impaired the methylation of *ECEL1* serving as an H3K27me3 modification site, which led to the dysfunction of the second topological domain. Therefore, we concluded that the compound heterozygous mutation caused the pathogenic phenotype of this proband.

**Conclusion:**

The present case highlights the usefulness of molecular genetic screening in diagnosing unexpected joint disorder. Identification of novel mutations in the *ECEL1* gene broadens the mutation spectrum of this gene and adds to the genotype-phenotype map of DA5D. Furthermore, rapid whole-exome sequencing analysis enabled timely diagnosis of this rare disease, facilitating appropriate treatment and scheduled follow-up to improve clinical outcomes.

## 1 Introduction

Distal arthrogryposis is a congenital contracture disorder that is inheritable. The disorder mainly affects the flexion and extension of distal joints and is not associated with neurological and/or muscle diseases. Since it was introduced by Hall et al. (1), several clinical manifestations have been described, including contractures of distal joints, scoliosis, sensorineural hearing loss, ophthalmoplegia, multiple pterygium, and camptodactyly. The contractures of distal joints, such as those in the ankle, knee, hip, hand, wrist, elbow, and shoulder, typically contribute to the significant morbidity of all subtypes of distal arthrogryposis. Based on the typical clinical presentations, distal arthrogryposis can be divided into several subtypes (2). Distal arthrogryposis type 5D (DA5D) is a rare genetic disorder that primarily affects the hands and feet, resulting in multiple joint contractures, muscle weakness, and bone abnormalities. In addition, DA5D reveals a unique feature due to its specific extraocular muscle involvement, leading to ophthalmoplegia and ptosis (3). About 50% of distal arthrogryposis cases are caused by genetic variants that encode skeletal myofibers’ contractile proteins, including *TPM2*, *TNNI2*, *TNNT3*, *MYH3*, *MYBPC1*, *MYH8*, *FBN2*, *PIEZO2* and *ECEL1* (3). Currently, there is insufficient research to fully elucidate the genetic-related molecular function of the reported gene, while most of the diseases demonstrate autosomal recessive inheritance. Genome sequencing has been used to identify a missense mutation in the *MYH3* gene in a family with DA5D. *MYH3* encodes a myosin protein involved in muscle contraction, and the mutation was found to disrupt the protein’s function, leading to abnormal muscle development and contractures (4). Similarly, a study identified a *de novo* missense mutation in the *PIEZO2* gene, which encodes a mechanosensitive ion channel involved in touch and proprioception. The mutation was found to affect the function of the channel, leading to abnormal sensory feedback and joint contractures (5). In addition, a mutation was identified in the *GPR126* gene in a family with DA5D. *GPR126* encodes a transmembrane protein involved in myelination and nerve function, and the mutation was found to affect the protein’s function, leading to abnormal development of the peripheral nervous system and joint contractures (6). Identifying these additional mutations may also provide new insights into the pathogenesis of the disease and potential therapeutic targets.

Endothelin-converting enzyme-like 1 (*ECEL1*) is a transmembrane zinc metalloprotease primarily localized in the endoplasmic reticulum. It is known to be involved in developing neuromuscular junctions and bone development during the prenatal phase (7, 8). *ECEL1* had been confirmed to be involved in the bone development and chondrocyte homeostasis. Through a pan-genomic approach, Dieterich et al. confirmed that homozygous or compounded heterozygous variants in the *ECEL1* gene on chromosome 2q37 were associated with DA5D. The disorder is caused by mutations impairing the function of ECEL1, resulting in abnormal development of the neuromuscular junctions and synapses, leading to cartilage degradation. However, there are only a few reported cases of DA5D associated with ECEL1 variants, which highlights the need for further understanding of ECEL1’s molecular function.

In the present study, we reported a six-month-old male infant who suffered DA5D, and a compound heterozygous mutations of *ECEL1* (NM_004826: c.2152-15C>A, c.110_155del p.F37Cfs*151) had been identified in the proband. Furthermore, the allele of c.2152-15C>A was maternally inherited, and the allele of c.110_155del was paternally inherited, a novel pathogenic variant of *ECEL1*. This report expanded the understanding of *ECEL1* in DA5D and emphasized the importance of assessment of ECEL1 compound heterozygous variants in patients with joint disorder. Also, the timely WES detection promoted early intervention for DA5D which help to improve the prognosis.

## 2 Methods

The study was approved by the ethics committee of the West China Second Hospital of Sichuan University (approval number 2014-034). In addition, we obtained written, informed consent from the patient’s parents prior to performing WES and for the inclusion of the patient’s clinical and imaging details in publications.

The genetic test had been performed at 8 months-old. The peripheral blood sample was obtained from the patient in an ethylenediaminetetraacetic acid (EDTA) anticoagulant blood sample tube that stored at 4°C for less than 6 h. DNA was extracted using the Blood Genome Column Medium Extraction Kit (Tiangen Biotech, Beijing, China) according to the manufacturer’s instructions. WES was performed using the NovaSeq 6000 platform (Illumina, San Diego, CA, United States), and the raw data were processed using FastP to remove adapters and filter low-quality reads. Paired-end reads were aligned to the Ensembl GRCh37/hg19 reference genome using the Burrows–Wheeler Aligner. Variant annotation was performed in accordance with database-sourced minor allele frequencies (MAFs) and practical guidelines on pathogenicity issued by the American College of Medical Genetics. The annotation of MAFs was performed based on the 1,000 Genomes, dbSNP, ESP, ExAC, and Chigene inhouse MAF database, Provean, Sift, Polypen2_hdiv, and Polypen2_hvar databases using R software (R Foundation for Statistical Computing, Vienna, Austria).

## 3 Case presentation

### 3.1 Clinical presentation and physical examination

**Figure 1:**
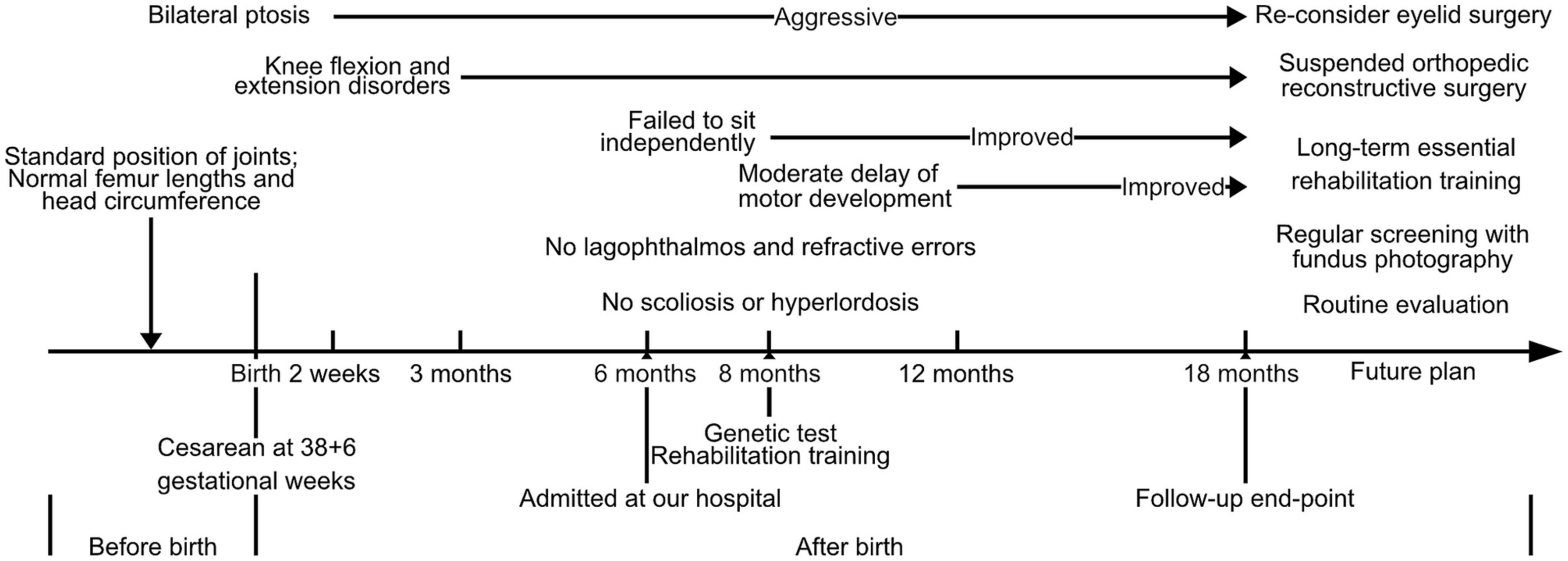
Time-line diagram presented the onset timepoints of major symptoms and further therapeutic plan for this DA5D patient with a compound heterozygous variant of *ECEL1* (c.2152-15C>A and c.110_155del).

The proband was a male infant admitted to our hospital at 6 months of age. However, the patient had received several medical consultations starting from 1 month of age due to severe and worsening symptoms of bilateral knee flexion and extension disorders and bilateral ptosis. In addition to the two primary symptoms, both thumbs showed adduction. and the facial abnormalities were arched eye brows, micrognathia, broad frontotemporal and high palatine arches. The parents provided gestational information that the routine fetal assessment ultrasound had identified the patient’s standard position of major joints, while femur lengths were within the normal range at different gestational stages. In addition, cranial screening and echocardiography found no impairments or abnormalities. No fetal genetic tests related to low risk of Down syndrome were conducted. The proband was delivered by cesarean section at 38 + 6 gestational weeks, with a birth weight of 2,600 g (P3.6), height of 50 cm (P41.6), and head circumference of 34 cm (P33). Initial physical examination after birth showed fully extended joint movement and normal muscle tone. Furthermore, this was the first child of the couple, and they denied any positive family history of chromosomal abnormalities, birth defects, autoimmune disease, rheumatoid arthritis, epilepsy, and neurological developmental disorder. Additionally, they denied any potential exposure to teratogens during pregnancy. All onset time points of major symptoms are presented in a timeline diagram in Figure 1.

**Figure 2:**
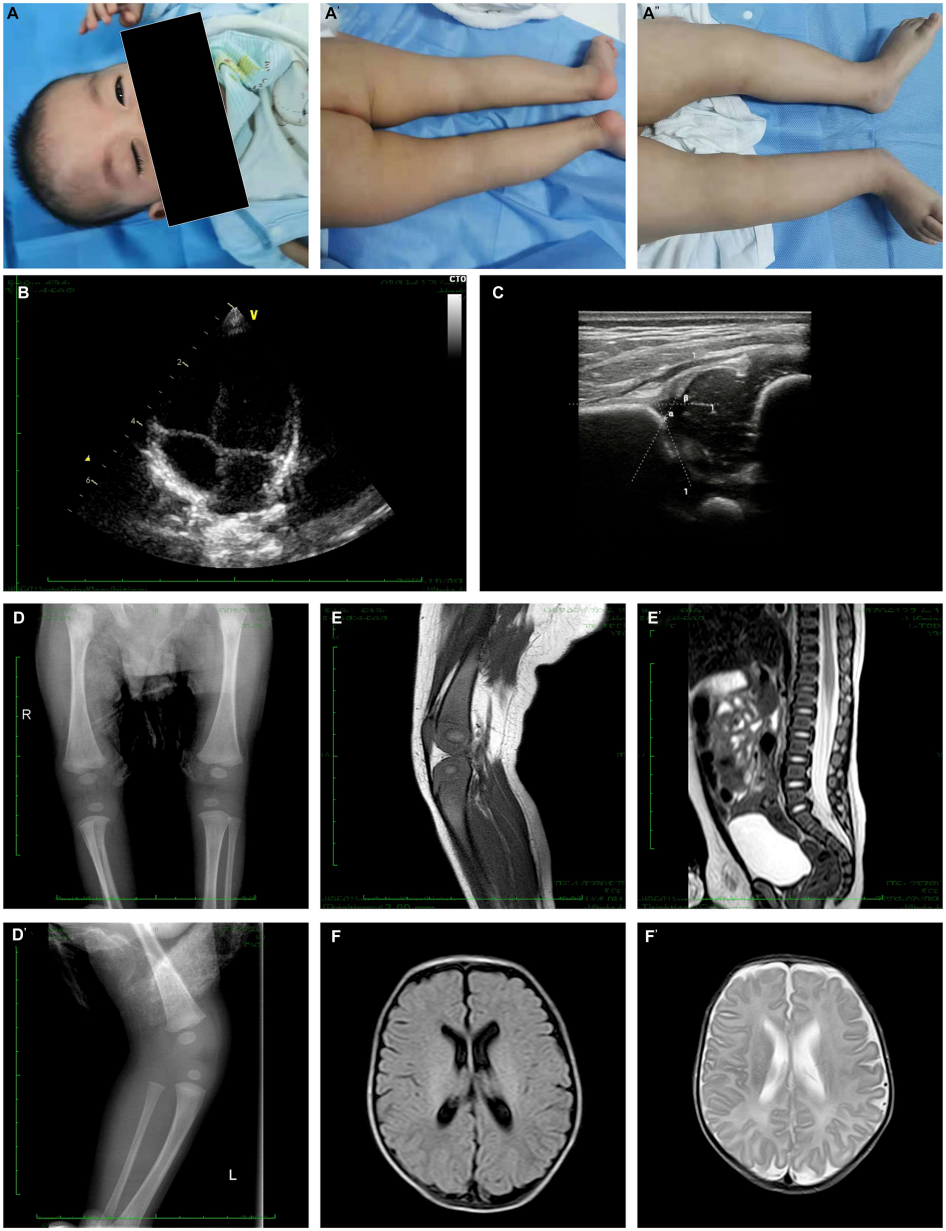
Clinical and radiology manifestation in the current proband. **(A)** The proband presented bilateral ptosis and impaired flexion of bilateral knees. **(B)** Echocardiography demonstrated a normal heart function and structure. **(C)** Hip ultrasonography showed Graf type I structure on his left hip joint. **(D)** X-ray examination of hip joint and knee joint showed that there was no definite abnormality in anterior-posterior view, while the lateral view demonstrated an over-extensive positive of knee joint. **(E)** The joint MRI presented a normal signaling in muscles, bones and ligaments associated to his knee joints while it still presented an over-extensive position, and the tail vertebras were normal. **(F)** The cerebral MRI revealed a normal structure and signaling both in T1 and T2.

According to the patient’s parents, bilateral ptosis was first observed at 2 weeks of age (Figure 2A). The ophthalmological evaluation revealed myogenic ptosis with levator palpebrae dysfunction. Lagophthalmos and refractive errors were not detected at such a young age. Additionally, the patient’s parents reported that bilateral knee flexion disorders began at 3 months of age (Figures 2A′–A′′). At this age, the patient’s weight, length, and head circumference percentiles remained between P20 and P50.

No other birth defects were detected based on the physical examination performed when the patient was 6 months old. The skull shape was within normal limits, and visual and hearing evaluations were normal. Physical examination of the chest, heart, and abdomen did not reveal any abnormalities, except for the absence of scoliosis or hyperlordosis. The patient’s motor development was assessed and found to be expected for neck, upper limb, and foot movements, with the ability to maintain balance while sitting. However, both knees showed impairments in both flexion and extension. Specifically, the left knee could only be passively flexed at an angle of 50°, while the right knee could be passively flexed to a degree of 55°, and neither joint could be placed in an extended position. In addition, external rotation of the hip joint was slightly impaired, which limited the movements of the lower extremities. The spine was normal without any scoliosis or curvature.

At the age of 8 months, the patient exhibited aggressive and severe ptosis. Furthermore, at 8 months old, he was unable to sit independently and failed to handle grasp building blocks by 12 months of age. The Gesell evaluation conducted at 12 months revealed a moderate delay in gross and fine motor development. Consequently, the proband underwent regular daily rehabilitation training, including head control, rolling over, crawling training, assisted standing, bilateral hand grasping, and sitting balance, as part of the training program.

### 3.2 Laboratory and imaging evaluation

Blood gas analysis, blood cell counts, and hepatic and renal function yielded no significant findings. The essential metabolic screening did not identify any impairment. The echocardiography demonstrated a normal heart function and structure (Figure 2B). The Hip ultrasonography showed a Graf type I structure on his left hip joint and a Graf type IIa structure on his right hip joint (Figure 2C). X-ray examination of the hip and knee joint showed no definite abnormality in the anterior-posterior view. In contrast, the lateral view demonstrated an over-extensive positive of the knee joint (Figures 2D,D′). In addition, the joint MRI presented normal signaling in muscles, bones, and ligaments associated with his knee joints while it still presented an over-extensive position with a decreased volume of cartilage, indicating degradation of chondrocyte, while the tail vertebras were normal (Figures 2E,E′). The cerebral MRI revealed a typical structure and signaling in T1 and T2 (Figures 2F,F′). Moreover, electromyography was involved, which presented a normal muscle response.

### 3.3 Molecular results

**Figure 3:**
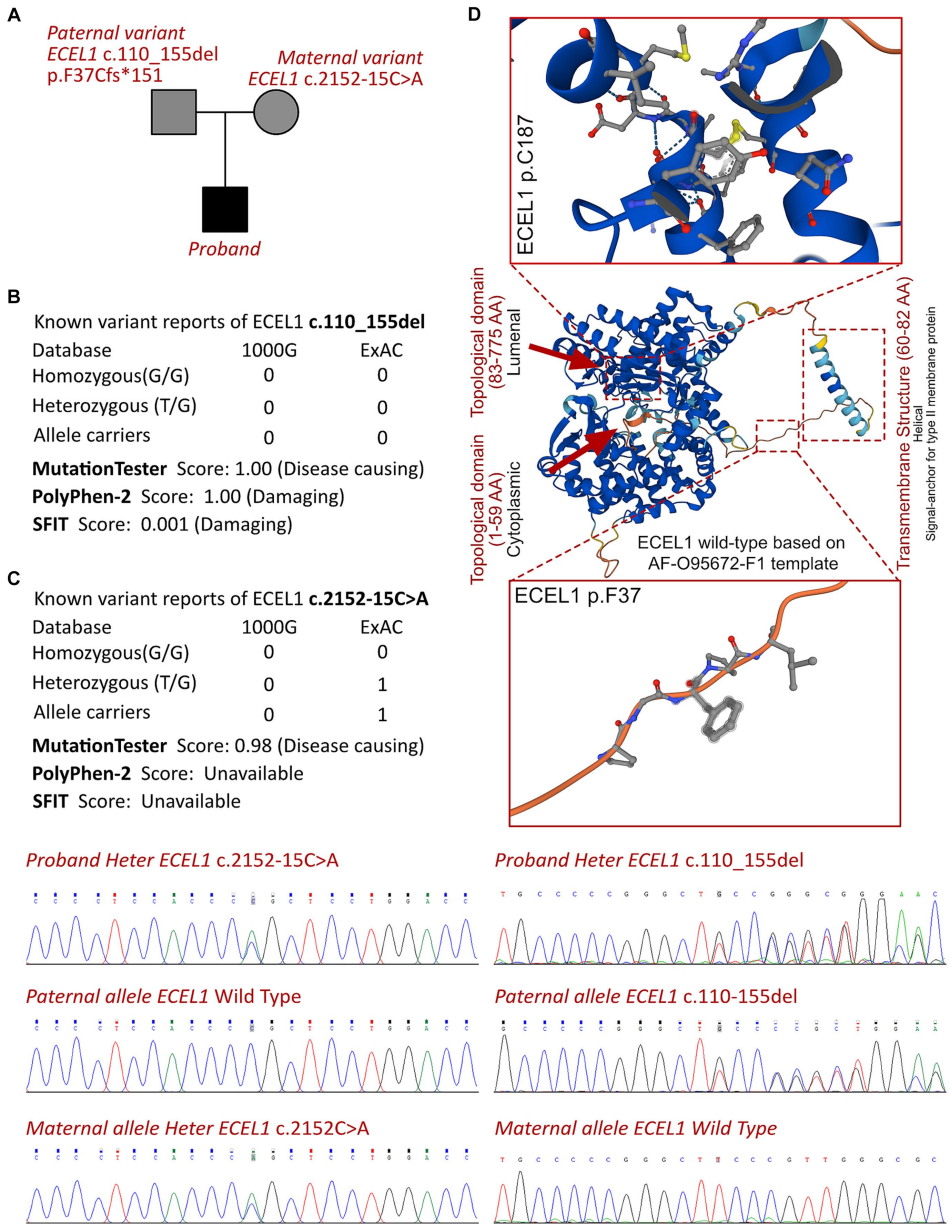
The ECEL1 molecular analysis. **(A)** The proband exhibited a compound heterozygous variant of ECEL1 (c.2152-15C>A and c.110_155del, p.F37Cfs*151). **(B)** The variant of ECEL1 c.110_155del had never reported in 1000G and ExAC, it has predicted protein damaging by PolyPhen-2 and SFIT, while only one report had been retrieved of the ECEL1 c.2152-15C>A variant. **(C)** Sanger sequence validation. **(D)** Protein structure predicted by AlphaFold (AF-O95672-F1), two topological domains and one transmembrane structure had been revealed with analyzed crystal structure. The variant of c.110_155del leads to the truncated protein locating in the substructure of transmembranous site.

According to the analysis result of WES, a novel compound heterozygous variant had been identified as c.2152-15C>A and c.110_155del (p.F37Cfs*151) of *ECEL1* gene. The Sanger validation demonstrated that the c.2152-15C>A allele was maternal inherited and the c.110_155del allele was paternal inherited (Figure 3A). The variant of *ECEL1* c.110_155del had never reported in database (Figure 3B), while two cases reported the variant of *ECEL1* c.110_155del. However, this should be the first report of a patients with the compound variants with c.2152-15C>A and c.110_155del together. The Sanger sequencing had been presented in Figure 3C. Besides, we had excluded all the potential variants involved in neurological development and muscle disordered. Then we reviewed all the other variants which were reported as pathogenic or likely pathogenic ones, and none of them was confirmed to be associated with the phenotype of the proband. So that, we suspected the compound heterozygous variant of ECEL1 contributed to the pathogenic phenotype of this proband. To elucidate the molecular architecture of the human *ECEL1* gene, we used MutationTaster with R software to predict the pathogenicity of *ECEL1* c.2152-15C>A and c.110_155del (p.F37Cfs*151), and assess the impact of these mutations on protein structure. As there was no available full-length protein crystal structure for ECEL1 which had been analyzed by X-ray or cryo-EM, AlphaFold protein structure software^1^ tool had been used to predicted protein crystal structure. The protein structure of ECEL1 has been built and named AF-O95672-F1 (9, 10). Within the structure, two important domains (topological domain) had been revealed with analyzed crystal structure. Then we performed modeling analysis using the SWISS-MODEL^2^ for the domain in wild type with the AF-O95672-F1.A template. We estimated the capability of the protein structure using Ramachandran plots. According to the American College of Medical Genetics, the mutation c.110_155del has certain pathogenicity (PVS1 + PM3 + PM2_Supporting), while the mutation c.2152-15C>A has not been reported in any populations and has uncertain pathogenicity (PM2_Supporting+PM3). The analyses from MutationTaster revealed the variant of c.110_155del impaired the transcription of *ECEL1* leading to amino acid sequence changes, frameshift, protein features affected, and truncated proteins and the variant was predicted as disease causing. The truncated protein lost the substructure of transmembranous site (Figure 3D). Although the variant of c.2152-15C>A of *ECEL1* would not alter the amino acid sequence in prior of exon 16, but this variant impaired the methylation of *ECEL1* serving as a H3K27me3 modification site, which was also predicted as disease causing due to the potential dysfunction of second Topological domain. DA5D is caused by homozygous or compound heterozygous variants, especially for *ECEL1*, which contributes as an autosomal recessive manner. So that, the newly identified variants of *ECEL1* need to be addressed their pathogenicities in specific cases. Although the variants of *ECEL1* c.2152-15C>A and c.110_155del had been retrieved in databases, there was no reported diagnosed cases could be analyzed for each variant. It means, such variants had been observed in population sequencing screening, and there was no convinced evidence to make an association between the mutations of *ECEL1* c.2125-15C>A and c.110_155del. Thus, this is the very first case to present a certain diagnosed case of DA5D with compound heterozygous variants, indicating and verifying the pathogenicities of the two variants, and draw an association between the novel genotype and clinical phenotype. So that, we concluded that the compound variant of *ECEL1* would be responsible for the pathogenic phenotype of this proband.

### 3.4 Final diagnosis and treatment

Upon analyzing the clinical manifestations, conducting imaging assessments, and genetic screening, the patient has been diagnosed with DA5D. A long-term follow-up plan has been devised for the proband, which includes ophthalmological, orthopedic, neurological, and developmental evaluations. The ophthalmologists have suggested regular screenings with fundus photography for the proband and re-evaluating eyelid surgical treatment based on the examination. Although there were no lagophthalmos and refractive errors, previous studies have shown that such complications are age-dependent, and regular evaluation is helpful for early detection. Tarsorrhaphy would be considered during follow-up to minimize corneal exposure, which is the most adverse ophthalmic complication leading to corneal perforation and blindness. Treatment for ptosis should be provided when the visual axis is blocked.

Orthopedic assessments are critical in managing DA5D, and at 18 months of age, the degree of joint contractures of hip joints is not significantly severe. Therefore, orthopedic reconstructive surgery has not been performed. The general structure of the spine is typical, and other joints do not show any contractures. However, due to fibrosis proliferation, the contracture phenotypes are expected to limit the function of major joints more extensively, and the indication for surgical therapy should be reconsidered. The parents have been informed that such surgeries may not be avoidable to relieve joint contractures and scoliosis. Rehabilitation treatment of the hip joint has been performed to maintain its development. Long-term essential rehabilitation training has improved muscular function, enabling the proband to sit and walk independently, which was the perspective of the patient’s parents. Rehabilitation training includes three aspects: movement, language and environment adaptation training. The important exercise training for this patient includes exercises such as pinching objects with fingers, turning over, crawling, and walking with railings. However, the proband experiences significant difficulty in completing squatting. A re-examined Gesell assessment at 18 months old revealed only slight delays in gross and major motor movements, significantly improving compared to the situation before rehabilitation therapy. And the maximal passive flexibility degree of hip-joint would up to 120 degree. Therefore, regular and long-term rehabilitation therapy is still considered helpful and should be administered as early as possible, which would benefit the prognosis of this disease.

## 4 Discussion

Distal arthrogryposis is a specific type of arthrogryposis, and its symptoms typically involve restricted movement of the distal joint, which cannot be fully flexed or extended. These impairments can affect various body parts, such as the feet, ankles, knees, hands, wrists, elbows, hips, and shoulders. In some severe cases, impairments in temporomandibular and atlantoaxial joints may also be present since birth. The rapid development of genetic, molecular, and radiological imaging techniques has provided essential insights into distal arthrogryposis. Clinical features and genetic associations have contributed to the subtype’s identification. However, since distal arthrogryposis is a rare disorder, there is limited cohort research on this condition. Nevertheless, case reports have illustrated the essential clinical characteristics of distal arthrogryposis. During fetal development, reduced intrauterine movement may accumulate connective tissue around the distal joints, resulting in a pathogenic phenotype postnatally (11). With the increased identification of genetic variants in this condition, 10 gene variants have been associated with distal arthrogryposis, including *TPM2*, *TNNI2*, *TNNT3*, *MYH3*, *MYBPC1*, *MYH8*, *FBN2*, *PIEZO2*, and *ECEL1* (3). These genes play crucial roles in cartilage development, myofibroblast differentiation, and proliferation. At present, this disease is considered to be a congenital non-progressive disease. For children with early detection and diagnosis, rehabilitation training can significantly improve related joint symptoms in the critical period of their development, and later surgical treatment is more of a supplementary treatment for patients with severe related symptoms and who can accept surgery.

Previous literature reviews have shown that ptosis is the most commonly observed clinical manifestation in patients with DA5D, with 43 out of 57 reported cases experiencing ptosis (3, 8, 12–26). This characteristic is considered congenital and myogenic and results from the lack of an eyelid crease and dysfunction of the levator muscle (27). Fibrosis of the muscle can also cause significant ocular complications, including lagophthalmos and corneal injuries. Aggressive contractures, including scoliosis, can also result from joint fibrosis. While motor function development is often delayed in younger patients, most of them exhibit nearly regular motor function (20, 22). Therefore, early diagnosis, careful follow-up to manage related complications, and administering rehabilitation therapy are crucial to treating this disease. However, ptosis or limited hip abduction may not be specific symptoms in children, which can lead to delayed diagnosis of DA5D. Additionally, the molecular mechanisms of DA5D remain unclear, which hinders the development of precise medications. With the rapid application of WES in diagnosing rare diseases, DA5D can be identified more definitively in a shorter time, providing more molecular information about the etiology of these diseases. Foe this proband, the variant of *ECEL1* c.110_155del could cause the truncated protein which lead to the transcriptional and translational impairments. Given that, it was critical to identify the impact of *ECEL1* c.2125-15C>A on protein structure and function. Indeed, the variant hind in splice site might not result in protein structural formation. Otherwise, it would significantly reduce the expression of *ECEL1*, as DA5D was associated with autosomal recessive manner which indicated the bi-alleles dysfunction. According to the analysis and the convinced clinical manifestation and targeted variants, we assumed that the variant of ECEL1 c.2125-15C>A in splice site would impair the translation of ECEL1.

DA5D is a specific type of distal arthrogryposis inherited in an autosomal recessive manner and is characterized by the involvement of the extraocular muscles and cartilage. In this review, we examined case reports of DA5D from the past few decades and summarized all the genetic mutation sites. Their corresponding changed amino acid sequences and all the clinical features demonstrated by genetic manners (see Table 1). Dieterich et al. (8) conducted a genome-wide single-nucleotide polymorphism (SNP) genotyping association study and identified a relationship between DA5D and the *ECEL1* gene. The *ECEL1* gene contains 18 exons that encode UTRs and protein-coding-sequence domains, as well as three Zn^2+^ binding sites and two active sites called topological domains. ECEL1 cleaves neuropeptides at a specific site within the C-terminal region, and this cleavage is regulated by zinc binding to the protein. This study provides insight into the substrate specificity and regulation of ECEL1 (24). Dysfunction of the ECEL1 molecule can be caused by impairments in each domain and transmembranous part. The mutation disrupts the splicing of the *ECEL1* gene, resulting in a truncated protein that lacks key functional domains, leading to DA5D. Several studies have investigated the molecular role of ECEL1 in the nervous system. This study highlights the genetic and clinical heterogeneity of DA5D and expands the known spectrum of ECEL1 mutations associated with the disorder. *ECEL1* is also involved in processing the neuropeptide substance P, a crucial modulator of pain perception. Researchers have found that ECEL1 cleaves substance P at a specific site, producing a shorter, biologically active form of the neuropeptide (28). The researchers also found that ECEL1 cleaves these precursors at specific sites, producing a range of biologically active neuropeptides. In addition, several studies have implicated ECEL1 in regulating synaptic transmission and neuronal plasticity. For example, a study showed that ECEL1 regulates glutamatergic transmission in the hippocampus, a brain region involved in learning and memory (29). Furthermore, the researchers found that ECEL1 is involved in processing the neuropeptide somatostatin, which modulates the release of glutamate from presynaptic terminals. *ECEL1* mutations have been associated with the rare genetic disorder DA5D, characterized by multiple joint contractures and muscle weakness in the hands and feet. Interestingly, according to the reported cases, most patients presented with significant symptoms since birth, and the onset time did not differ between heterozygous variants and bi-allelic or homozygous variants. However, the difference between single-allelic and bi-allelic variants would determine the therapeutic strategy and the age at which surgery is performed. Unlike other subtypes of DA, which are inherited through autosomal dominance, DA5D is a rare disease that is inherited in an autosomal recessive manner. After the studies on the relationship between *ECEL1* and DA5D reported by McMillin (3) and Dieterich (8), almost all the subsequent studies on this disease have been related to *ECEL1*. Due to its rarity, no population-specific or race-specific associations have been reported. From the reported cases, there have been no reports of fetal death, but there are significant developmental disorders. Meanwhile, some patients who were not diagnosed and intervened in time after birth will die due to complications caused by related malformations, such as death caused by the influence of breathing related muscles and chest malformations. We believe that this disease is a birth defect, and the reported patients almost all showed corresponding typical symptoms after birth. The difference in mutation sites is more likely to lead to the difference in severity of symptoms. The influence of other exposure factors is still unclear.

**Table 1:** Summary of reported *ECEL1* mutations and clinical features.

Reference	Nucleotide (cDNA)	Protein alteration	Distal arthrogryposis	Ocular phenotype	Other manifestation
McMillin et al., Am J Hum Genet. 2013	c.716dupA (exon2)	p.Tyr239* (*n* = 2)	Onset age: 1 year Contractures of foot, ankle, knee (flexion), hip, hand, wrist, elbow and shoulder	Right ptosis	Bulbous nose Scoliosis Mild micrognathia
	c.716dupA c.344_355 del (exon2)	p.Tyr239* (*n* = 1)	Contractures of ankle, knee (flexion), hip, hand, wrist, elbow, shoulder and neck	Right ptosis	Bulbous nose Reduced facial expression Slightly crouched gait Pterygia in neck and axillae Micrognathia
	c.869A>G (exon4) c.797_801delins GCT (exon3)	p.Tyr290Cys p.Asp266Glyfs*15 (*n* = 2)	Contractures of foot, ankle, knee (extension), hip, hand and wrist	One is right ptosis; another one is mild bilateral ptosis	Bulbous nose Mild micrognathia Short neck Glabellar hemangioma Posteriorly rotated ears
	c.1252C>A (exon7) c.1184-3A>T (exon6)	p.Arg418Ser (*n* = 2)	Contractures of foot, ankle, knee(extension), hip, hand and wrist	One is right ptosis Another one is normal	Bulbous nose Micrognathia
	c.1184G>A (exon6)	p.Arg395Gln (*n* = 1)	Contractures of foot, ankle, knee (extension), hip, hand, wrist, elbow, shoulder and neck	Right ptosis	Bulbous nose Pterygia in elbows, axillae and neck Dislocated hips Reduced fetal movements
	c.590G>A (exon2) c.1252C>T (exon7)	p.Gly197Asp p.Arg418Cys (*n* = 1)	Contractures of foot, ankle, hip, hand, wrist, elbow, shoulder and neck	Severe bilateral ptosis	Bulbous nose Micrognathia Short neck Cupped ears
Dieterich et al., Hum Mol Genet. 2013	c.1649C>G (exon10)	p.Ser550* (*n* = 2)	Flexed fifingers III–V Congenital hip dislocation and/or limited hip movement Limited knee flexion Talus or talus valgus deformity of feet	Ptosis Pseudoexophthalmos and lagophthalmos	Reduced facial expression Tongue atrophy Speech difficulties Short neck Scoliosis Hyperlordosis
	c.1470G>A (exon8) c.997C>T (exon5)	p.Try490* p.Arg333* (*n* = 1)	Flexed fifingers III–V Distal interphalangeal joint hyperlaxity Congenital hip dislocation and/or limited hip movement Limited knee flexion Talus or talus valgus deformity of feet	—	Tongue atrophy Short neck Scoliosis Hyperlordosis
	c.874delG (exon4)	p.Val292Cysfs*51 (*n* = 1)	Flexed fifingers III–V Congenital hip dislocation and/or limited hip movement Limited knee flexion	Ptosis	Small mouth Short neck
	c.1685+1G>T (intron)	p.Lys552AlafsX33 (*n* = 2)	Flexed fifingers III–V Distal interphalangeal joint hyperlaxity Congenital hip dislocation and/or limited hip movement Limited knee flexion Talus or talus valgus deformity of feet	Ptosis	Short neck Hyperlordosis
	c.966+1G>A (intron)	p.Asp559AlafsX33 (*n* = 1)	Flexed fifingers III–V Limited knee flexion Talus or talus valgus deformity of feet	Ptosis	Mouth held open Short neck Sucking and swallowing difficulties Scoliosis Hyperlordosis
	c.2278C>T (exon18)	p.Cys760Arg (*n* = 3)	Flexed fifingers III–V Congenital hip dislocation and/or limited hip movement Limited knee flexion Talus or talus valgus deformity of feet	Only one with ptosis	Tongue atrophy Short neck Scoliosis Hyperlordosis
Khan et al., J AAPOS. 2014	c.1221_1223dup (exon7)	— (*n* = 4)	Contractures of the hands and feet	Three of 4 have strabismus with abnormal synkinesis and ptosis (one is bilateral and other two are unilateral)	—
Shaheen et al., Clin Genet. 2014	c.1221_1223dup (exon7)	— (*n* = 4)	Camptodactyly	Three with ptosis and Strabismus	Two with scoliosis Short stature
	c.1057dupC (exon5)	— (*n* = 2)	Camptodactyly Hip dislocation	Ptosis	—
	c.1210C>T (exon7)	p.Arg404Cys (*n* = 3)	Camptodactyly Club foot Hip dislocation	One with ptosis	—
	c.1819G>A (exon13)	p.Ser607Gly (*n* = 2)	Contractures of hand, knee (flexion), wrist and shoulder	Ophthalmoplegia One with ptosis	Arched eye brows Small mouth Short neck Reduced facial expression Bulbous nose Micrognathia Scoliosis One with hyperlordosis
Barnett et al., Am J Med Genet A. 2014	c.1531G>A (exon9) c.1797-1G>A (intron 12/exon 13 boundary)	p.Gly511Ser (*n* = 2)	Contractures of foot, ankle, knee, hip, hand, wrist and elbow One with neck Contractures	Ptosis	Micrognathia Pterygia Central tongue atrophy/grooved tongue
Patil et al., Am J Med Genet A. 2014	c.2023G>A (exon15)	p.Ala675Thr (*n* = 1)	Limitated in knee flexion and elbow movement Hands with camptodactyly and/or ulnar deviation of fingers Congenital dislocation of hips	Ptosis Refractive errors Light pigmented fundus	Bulbous nose Micrognathia Small mouth Cleft palate Furrowed tongue Pterygia Short neck Hypoplastic labia majora Scoliosis
Bayram et al., J Clin Invest. 2016	c.1147C>T (exon6)	p.Gln383X (*n* = 2)	One with severe flexion contracture of knees, camptodactyly, hip dislocation The other one with contractures of hands, hip-joint dysplasia	—	The other one with a mask-like whistling appearance, and short neck
Stattin et al., Am J Med Genet A. 2018	c.1163 T>C (exon6)	p.Leu388Pro	Contractures of wrist, shoulder, ankle, knee, metacarpophalangeal joints(extension); hip-joint dysplasia	Unilateral ptosis	Reduced facial expression Lacrimal duct stenosis Bulbous nose Small mouth
Ullmann et al., Neuromuscul Disord. 2018	c.589G>A (exon 2)	p.Gly197Ser (*n* = 1)	Contractures of finger, shoulder, elbow, wrist, hip, ankle and knee	—	Reduced foetal movements Breech presentation Cleft palate Central groove in tongue Micrognathia
	c.2005_2006delAC (exon 15)	p.Thr669fs (*n* = 1)	Contractures of finger, shoulder, hip and knee	Bilateral ptosis	Reduced foetal movements Breech presentation Central groove in tongue Neck webbing Scoliosis
	c.1470G>A (exon8)	p.Trp490Ter (*n* = 2)	Contractures of finger, ankle and knee	One with bilateral ptosis	Breech presentation One with micrognathia
Umair et al., Front Pediatr. 2019	c.158C>A (exon 2)	p.Pro53Leu (*n* = 2)	Contractures of finger, elbow, ankle and knee Camptodactyly	Ptosis Strabismus	Facial dysmorphism
Jin et al., Biomed Res Int. 2020	c.69C>A (exon 2) c.1810G>A (exon 13)	p.Cys23Ter p.Gly604Arg	Bilateral contractures of the fingers, wrist, elbow, and knees	Left ptosis	Webbing of the bilateral fingers and elbows Arched eyebrows, strabismus, protruding ears, and cleft palate
Alesi et al., Int J Mol Sci. 2021	c.1507-9G>A (intron)	— (*n* = 2)	Contractures of elbow and knee	Unilateral ptosis	Decreased fetal movements Congenital hip dislocation
Huddar et al., Children. 2021	c.602 T>C (exon2)	p.Met201Thr (*n* = 1)	Multiple joint contractures	Asymmetric ptosis	Motor developmental delay Pes planus, kyphoscoliosis, undescended testis Distal lower limb weakness
	c.83C>T (exon2)	p.Ala28Val (*n* = 2)	Multiple contractures, pes cavus, prominent hyperextensibility at the knee, hypotonia of lower limbs, wasting and weakness of all limbs (distal > proximal)	Unconjugated eye movements, and primary optic atrophy	White hairlock Tented upper lip Bulbous nose Tongue furrowing Small low set ears
Zhang et al., Taiwan J Obstet Gynecol. 2021	c.110_155del46 (exon2) c.633G>C (enon2)	p.Phe37Cysfs*151 p.Trp211Cys	Prenatal ultrasound examination demonstrated reduced fetal movements, clenched hands, fixated extended knees; Rocker bottom feet and scoliosis	—	—
Gowda et al., Clin Dysmorphol 2021	c.493_517del (enon2)	p.Leu165Alafs*30 (*n* = 3)	Contractures of hip, hand, finger, elbow, ankle, wrist, shoulder, neck, foot and knee Talus valgus/Varus, abnormal foot curvature, short toes	Ptosis Pulled lower palpebra	Short stature Limited facial expression Facial asymmetry Arched eyebrows Bulbous nose Scoliotic spine
Cohen et al., Ophthalmic Genet 2023	c.110_155del (enon2)	p.Phe37Cysfs*151 (*n* = 3)	The upper and lower limbs in various degrees; camptodactyly and adducted thumbs	Down-slanting palpebral fissures Ptosis; inferior scleral show	Scoliosis Arched eyebrows Small mouth and furrowed tongue
Ahangari et al., Mol Genet Genomic Med 2023	c.535A>G (exon2)	p. Lys179Glu (*n* = 2)	Limited shoulder movement and elbow movement; severe contracture of fingers II–V; talus valgus, deformity of feet and difficulty in walking	Mild ptosis Limitation of ocular motility in the vertical direction	Decreased facial movements; speech difficulties

According to previous researches, abnormal biological force loading to chondrocyte would induce the degradation of cartilage, including RAP2/YAP signaling (30). Moreover, integrins, TGFBR1/2, TRPV4, PIEZO1/2 channels also mediating the mechontransduction in chondrocyte (31). The overtime exceeding pressure loading impaired the normal metabolic hemostasis and cartilage development, resulting in decreased volume of cartilage in particular joints. Importantly, the degradation of cartilage had been considered as non-reversible pathophysiological process (31). Thus, the early relief abnormal pressure loading was critical in managing such diseases. According to this issue, the essential timely surgical treatment would be much benefit to maintain cartilage development and functional performance among the disorders of joint, especially for such muscular dysfunctions and arthrogryposis. In this case, the positive joint treatment would be much helpful to improve his prognosis by efficient clinical and molecular diagnosis via MRI screening and WES analysis.

In the current case, the proband exhibited a typical clinical presentation, including bilateral contractures of knee flexion and ptosis, strongly suggesting the diagnosis of DA5D. To confirm the diagnosis, a WES was performed, which identified a specific ECEL1 pathogenic variant allele from the patient’s father and an uncertain pathogenic variant allele from his mother, enabling a diagnosis of DA5D based on mutation site and protein structure assessments. None of the proband’s parents exhibited disease manifestations related to distal arthrogryposis, thus indicating that the novel compound heterozygous variant was responsible for the disease manifestation. A case report by Zhang et al. (25) has reported a compound mutation of *ECEL1*, however, prenatal ultrasound examination demonstrated reduced fetal movements, clenched hands, fixated extended knees and rocker bottom feet and scoliosis, and the pregnant chose to terminate the pregnancy. But in our report, ultrasound during pregnancy showed no abnormalities. Cohen has reported a case only with c.110_155del mutation in *ECEL1* (12). Compared with our patient, both cases presented ptosis, thumb adduction, arched eyebrows and scoliosis. However, our patient only had lower extremity knee contracture, while the other one presented with abnormalities in both upper and lower joints. Moreover, our patients received rehabilitation treatment for joint related symptoms and achieved better symptom improvement due to the early intervention time. Unfortunately, the two patients reported by Cohen were diagnosed in middle age and focused on the description and treatment of eye muscle related symptoms. Mutations in multiple exons and introns of the ECEL1 gene have been reported to induce DA5D, while mutations in exon 2, such as c.716dupA, c.344_355 del, c.590G>A, c.589G>A, c.158C>A, c.69C>A, c.602 T>C, c.83C>T, c.633G>C, and c.110_155del, were primarily recorded in DA5D patients, suggesting that impairment of exon 2 transcription significantly contributes to DA5D (3, 19–21, 24, 25).

## 5 Conclusion

In summary, a comprehensive evaluation of early-onset distal arthrogryposis is crucial, and WES can provide valuable genetic information to help diagnose specific types of distal arthrogryposis. DA5D is associated with ECEL1 variants that exhibit autosomal recessive inheritance, and mutations in exon 2 are essential in the context of ECEL1-related DA5D. This study expands the spectrum of ECEL1 mutations and provides essential information for the genotype-phenotype map of DA5D. Furthermore, the prompt diagnosis of this rare disease through rapid WES analysis can facilitate appropriate treatment by inhibition degradation of cartilage, thereby improving clinical outcomes.

## Data availability statement

The datasets presented in this article are not readily available because of ethical and privacy restrictions. Requests to access the datasets should be directed to the corresponding authors.

## Ethics statement

The studies involving humans were approved by West China Second Hospital of Sichuan University (approval number 2014-034). The studies were conducted in accordance with the local legislation and institutional requirements. Written informed consent for participation in this study was provided by the participants' legal guardians/next of kin. Written informed consent was obtained from the individual(s), and minor(s)’ legal guardian/next of kin, for the publication of any potentially identifiable images or data included in this article.

## Author contributions

SJ: Conceptualization, Data curation, Formal analysis, Investigation, Methodology, Software, Visualization, Writing – original draft. MP: Investigation, Methodology, Software, Supervision, Validation, Writing – original draft. YHe: Data curation, Formal analysis, Investigation, Methodology, Writing – original draft. YHu: Methodology, Project administration, Supervision, Validation, Writing – review & editing. JL: Conceptualization, Investigation, Methodology, Project administration, Resources, Software, Supervision, Validation, Writing – review & editing. YL: Conceptualization, Formal analysis, Funding acquisition, Investigation, Methodology, Project administration, Software, Supervision, Validation, Visualization, Writing – review & editing.

## Footnotes

^1^ https://alphafold.ebi.ac.uk/

^2^ https://swissmodel.expasy.org/
